# Mr. President and Gentlemen of the Association

**Published:** 1856-09

**Authors:** 


Dr. Thomas W. Blatchford, of Troy, was the representation of the American Medical Association, at the recent meeting of the American Association for the Advancement of Science. We give his address on his introduction:

*Mr. President and Gentlemen of the Association*—Dr. Zina Pitcher, of Detroit, to whom it properly belongs to represent the American Medical Association in this learned assembly, regrets exceedingly that unforeseen contingencies had occurred to prevent his being present on this occasion. But he cannot regret it as much as I do. I know he expected fully to be with you, and calculated on much enjoyment and profit while mingling in your deliberations. He would have added an interest to the occasion which would have done honor to the body over which he presides. But since the duty very unexpectedly devolves upon me, one of its humblest members, I will endeavor to discharge it to the best of my ability. In behalf, then, of the American Medical Association, I am to tender to you the thanks of that body for the very polite invitation extended to them, through your local committee, to attend this present meeting, and to inform you that the invitation was most cordially accepted, and that a hearty "aye" resounded from every part of that large assembly when the question of acceptance was taken. And why was the invitation so heartily responded to, but because every one present felt glad that there were others beside themselves bent on the discovery and elucidation of truth? Both associations are searching after facts which shall better the condition of our race. They have no other object in view, however various the paths they take to obtain it. I consider the formation of associations of this character to have given a greater impetus to the progress of science than any other cause which has, perhaps, ever been put in operation. These associations have called into active, vigilant exercise ten thousand minds, which, but for them, would have remained inactive or satisfied with the discharge of merely routine duties. They have enabled men, by the personal intercourse they afford, to measure themselves, not by themselves to entire exclusiveness, but by others, and thus many learn how much less they really know than they once thought they did. They also constitute a bright era in the nineteenth century; and if what they have already accomplished is an earnest of what the future will reveal, may we not look for results which will cause more astonishment than anything which even the present century has yet witnessed? These associations, it will be perceived, differ widely from those of Europe in the last century, inasmuch as they open wide the door of admission, so that any one having an offering to make, can bring it with his own hands. There is no exclusiveness, except in being exclusively free from quackery. Poverty here is no barrier to admission, for it is well known that many of our brightest gems are in possession of no other gems than that of the mind, unless they have one in their better half. In the Medical Association, at Detroit, we had the honor to receive delegates from the Canadas, and a delegation of more noble bearing never graced an assembly. You must allow me to express my gratification at seeing the same advantage enjoyed by this association likewise. Is it not calculated to fill us with hope for the future, notwithstanding the dangers which threaten us from the upheavings of political and sectional animosities, when we see a deliberative body, in harmonious session, for such an object, in which national distinctions, state boundaries, and geographical divisions are all obliterated—buried—forgotten in the one sole object of searching after truth? That the two associations may always be found to harmonize and fraternize is my sincere desire. And now, in behalf of the body I have the honor to represent, I hereby extend to you, gentlemen of the American Association for the Advancement of Science, the right hand of fellowship, assuring you that we shall ever be found ready, with head, heart and hand, to second any measure calculated to promote the noble object we all have in view.

We are indebted to the *Ontario Repository*, published at Geneva, for the following intelligent account of the illness of Dr. H. A. Potter, of that town, and the death of his brother, of Prattsburgh, Steuben Co., from a surgical *poisoned* wound:

*Case of Poisoning from Animal Matter.*—The recent serious illness of Dr. H. A. Potter, of this village, should serve as a warning to physicians, surgeons, and nurses, or those having charge or care of patients suffering from extensive suppurating wounds.

The faets in relation to this case, as furnished us by the Dr. and his physician, Dr. E. R. Maxson, of Geneva, and corroborated, as we understand, by the counsel had in the case, are as follows:

Dr. H. A. Potter, of Geneva, an eminent surgeon, well known in this and and other states, had charge of his brother, Dr. John W. Potter, of Prattsburgh, Steuben county, N. Y., during an illness of about one week, which terminated fatally. This illness was supposed to have been caused by the absorption of matter by a slight wound upon the hand, in dressing an extensively suppurated wound; the local and constitutional symptoms being similar to those usually attending dissecting wounds; extensive suppuration having taken place previous to death.

During Dr. Potter's attendance of his brother, he passed his fingers into the abscess, which he had opened, for the purpose of ascertaining its extent; and also, otherwise exposed his hands to the matter which was discharged. A slight scratch on the outside of the third finger of the right hand, gave him some uneasiness for two or three days up to July 13th, when, while attending the funeral of his brother, the Dr. discovered a redness along the lymphatics of the fingers; and also, a red spot on the back of the hand; which rapidly spread and soon covered the whole hand and arm nearly to the shoulder, the whole becoming red, enormously swelled and exceedingly painful. The shock to the nervous system was very severe, threatening his life, even during the first twenty-four hours.

Up to the third day, the constitutional symptoms were desperate; the hand and arm evidently approaching gangrene, was only saved from mortification by active and well-directed general treatment, and free scarification of the hand.

At this juncture, when scarcely a hope of recovery existed, the local and constitutional symptoms became, in a measure, controlled or arrested; and, as free suppuration in the hand and arm commenced by the seventh day, the chances of life became far better; and now, July 28th, though there are four points in the hand and arm from which matter is discharging, the Dr. is convalescent, and will probably recover, and has a fair prospect of regaining the use of the affected hand, so essential to him as an operating surgeon.

We have seldom witnessed a case, in which so much anxiety has been felt by community, as in this; and we feel sure that nothing short of a judicious treatment, good management, and a kind Providence, could have averted the almost certain fatal tendency of the malady.

The local and constitutional symptoms in this case were similar to those usually following dissecting wounds; and as such effects sometimes follow the absorption of matter from the living subject as well as from the dead, too much care cannot be exercised by those exposed to matter from suppurating, putrid, or sloughing wounds.

